# Outcomes and Complications Following Chronic Patellar Tendon Repair: A Systematic Review

**DOI:** 10.7759/cureus.41713

**Published:** 2023-07-11

**Authors:** Alexander K Hahn, Carlo Coladonato, John J Corvi, Neel K Patel, John Hayden Sonnier, Fotios Tjoumakaris, Kevin B Freedman

**Affiliations:** 1 Orthopaedic Surgery, University of Connecticut, Farmington, USA; 2 Sports Medicine, Rothman Orthopaedic Institute, Philadelphia, USA; 3 Orthopaedic Surgery, Mount Sinai Hospital, New York, USA

**Keywords:** injuries, autograft, chronic, knee, patellar tendon rupture

## Abstract

The purpose of this systematic review is to report outcomes and complications following the reconstruction of chronic patellar tendon ruptures.

Four databases (Cochrane Database of Systematic Reviews, PubMed, Embase, MEDLINE) were searched from inception to July 2021. Inclusion criteria included articles that (1) analyzed outcomes and complications following chronic patellar tendon reconstruction (>4 weeks from injury to repair), (2) were written in English, (3) greater than five patients, and (4) a minimum 2-year follow-up. Exclusion criteria included (1) non-original research and (2) patellar tendon repair/reconstruction with prior total knee arthroplasty. Data on outcome metrics and complications were extracted from the included studies and reported in a qualitative manner.

Nine studies (number of patients = 96) were included after screening. Seven studies analyzed autograft reconstruction, and three of those seven studies analyzed reconstructions with additional augmentation. The remaining two studies evaluated reconstruction utilizing a bone-tendon-bone (BTB) allograft. Four of the autograft studies (n=40 patients) showed a range of post-operative mean Lysholm scores of 74-94. Additionally, four studies reported a post-operative extensor lag of 0-3°. Post-operative protocol for autograft studies included delayed motion and was either contained to a bivalved cast or a hinged knee brace for six weeks. The two allograft studies reported a range of mean Lysholm scores from 62 to 67, and each immobilized the leg in full extension until six weeks.

While chronic patellar tendon ruptures are a rare injury of the extensor mechanism, there are viable options for reconstruction. Overall, chronic patellar tendon ruptures reconstructed with both autograft and allograft will provide fair to good outcomes with low complication rates. Following surgery, immobilization for at least six weeks should be emphasized to protect the graft and optimize patient outcomes.

## Introduction and background

Patellar tendon ruptures, which result in a disruption of the extensor mechanism, can be devastating injuries significantly affecting quality of life if left untreated [1,2]. Patients usually present with symptoms of weakness, difficulty standing up from a seated position, difficulty ambulating up and down stairs, and frequent falls due to balance issues while walking [3]. They are commonly found in patients with chronic diseases such as renal failure and diabetes or in young athletes (<40 years old) [4]. Patellar tendon ruptures can be subdivided into three distinct sites with proximal avulsion of the tendon from the inferior pole of the patella being the most common due to the more than threefold greater strain seen here than at the midsubstance of the tendon. Alternatively, the other two locations for rupture include the midsubstance of the tendon and the avulsion of the patellar tendon from the tibial tubercle [5]. Defining patellar tendon ruptures as acute or chronic is related to time to repair/reconstruction. Acute patellar tendon tears typically refer to any tear that occurs within two weeks of the time of surgery. Patients can present later due to neglect, missed diagnoses, or alternative treatment [6]. Currently, it is debatable how best to fix these chronic injuries and which technique is optimal. In the case of patellar tendon ruptures, primary repair in the acute phase is the most advantageous for proper repair and healing. As time progresses from the initial injury, the patella retracts proximally and makes reapproximating the tendon difficult [7]. Therefore, there have been many strategies to reconstruct the patella to restore the extensor mechanism.

Most commonly, techniques such as autograft reconstruction with the semitendinosus and gracilis tendon or allograft reconstruction with the Achilles tendon have been used [7]. The technique associated with the use of soft tissue grafts typically involves tunneling into the mid portion of the patella and posterior to the tibial tuberosity allowing the graft to be passed through the patella and anchored onto the tibia, thereby restoring the continuity of the patella and tibia. To allow for proper fixation of the graft, different techniques can be used such as patellar and tibial tuberosity tunnels in addition to using sutures to secure the grafts or interference screws to fixate the graft into bone [8]. Successful graft placement and fixation are of the most importance; however, the post-operative protocol plays an important role in graft protection and healing. Post-operative protocols following chronic patellar tendon rupture repair usually involve six weeks of immobilization in a bivalved cast or hinged knee brace to prevent graft pullout. After six weeks, patients usually start a passive range of motion (ROM), then slowly progress to an active ROM to reduce quadriceps atrophy [9,10]. After this period, full knee ROM is permitted with special attention by the patient to not perform any forceful eccentric contractions [10].

Currently, there is no consensus on which techniques are optimal for chronic patellar tendon rupture reconstruction given the paucity of outcomes data available in the literature [11]. Therefore, the purpose of this systematic review is to report outcomes and complications following the reconstruction of chronic patellar tendon ruptures. We hypothesized that both autograft and allograft reconstruction of chronic patellar tendon ruptures will result in similar outcomes with a low complication rate.

## Review

Materials and methods

Comprehensive Search Strategy

A review of the literature analyzing outcomes and complications following surgical reconstruction of chronic patellar tendon ruptures was performed in July 2021 using the Cochrane Database of Systematic Reviews, the Cochrane Central Register of Controlled Trials (1980-2021), PubMed (1980-2021), MEDLINE (1980-2021), and Embase (1980-2021) according to the PRISMA statement [12]. The following search terms were included and combined with the Boolean operators AND/OR: “patellar tendon,” “patella tendon,” “rupture,” “injury,” “repair,” “chronic,” “reconstruction,” “operative,” “surgery.” Duplicates were removed from the resulting studies. Inclusion criteria included articles that (1) analyzed outcomes and complications following chronic patellar tendon reconstruction (>4 weeks from injury to repair), (2) were written in the English language, (3) included more than five patients in the study, (4) followed patients for a minimum of two years, and (5) included human subjects. Exclusion criteria included (1) review articles, (2) cadaver studies, (3) biomechanical studies, (4) animal studies, and (5) patellar tendon repair/reconstruction in patients that previously underwent total knee arthroplasty.

Study Screening and Assessment of Study Quality

Two independent reviewers (AKH, CC) used the inclusion and exclusion criteria to screen the articles first by title, then by abstract, and then by full text. Any discrepancies between the reviewers were advanced to the full-text stage to avoid inadvertent exclusion. Any discrepancies at the full-text stage were resolved by discussion with a senior reviewer. Study quality and risk of bias were assessed by the same two reviewers using the methodological index for non-randomized studies (MINORS), which is a validated tool designed to quantify the methodological quality of non-randomized studies [13]. The final MINORS score for each study was calculated by averaging the scores of each reviewer.

Data Collection

The two reviewers (AKH, CC) independently extracted data from each study into pre-determined tables in Microsoft Excel (Microsoft, Washington, USA). Variables for analysis included cohort demographics, length of follow-up, surgical technique, complications, re-ruptures, functional outcomes, post-operative immobilization and rehabilitation, and post-operative range of motion (ROM). Means, standard deviations, and 95% confidence intervals were collected based on reporting of individual studies. The level of evidence for each study was assigned using the classification proposed by Wright et al. [14]. Due to significant heterogeneity in reporting, outcome measurements used, and reconstruction methods, a meta-analysis was unable to be performed. Therefore, all the aforementioned articles were reviewed qualitatively, and descriptive statistics, such as ranges, were used to combine abstracted data.

Results

Study Selection

**Figure 1:**
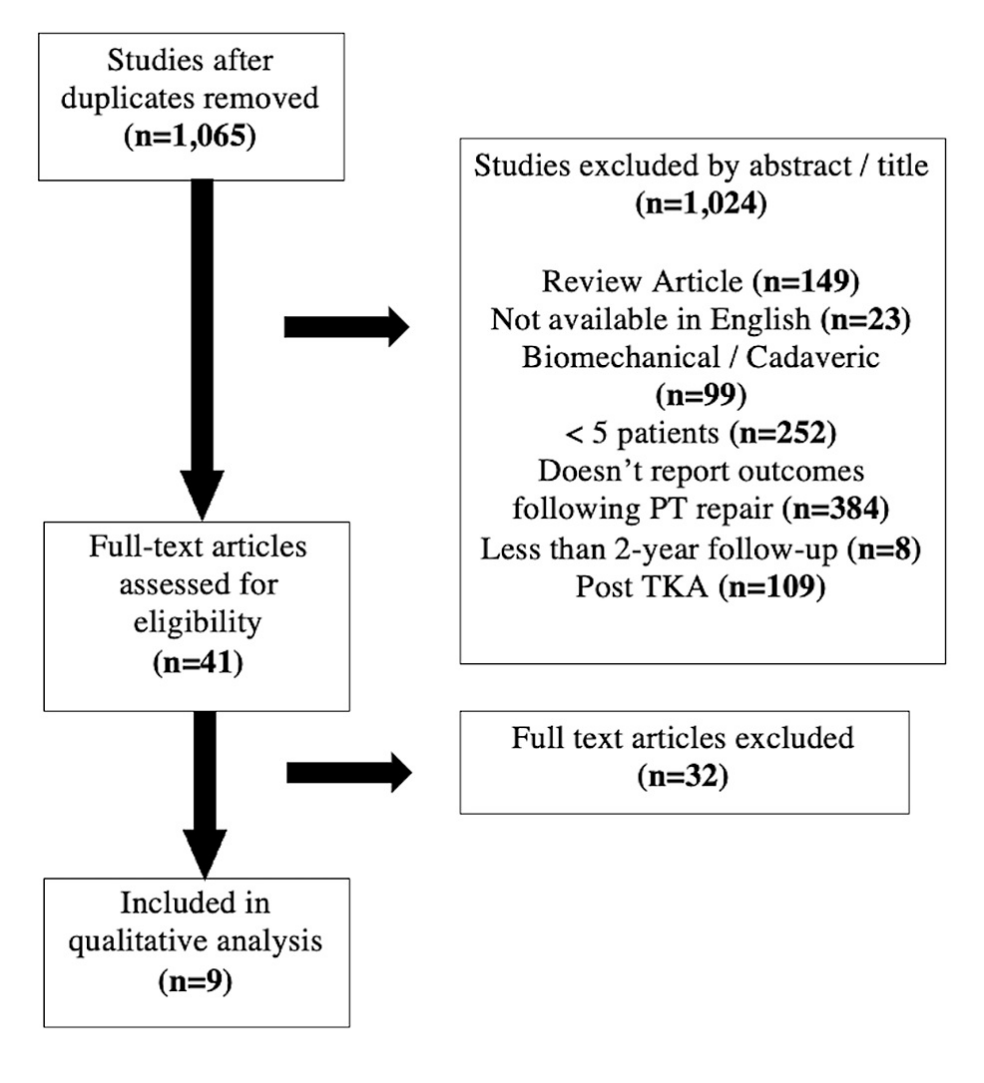
PRISMA flow chart for article selection PT: patellar tendon, TKA: total knee arthroplasty

A total of 1,065 studies resulted from the initial search of the databases after duplicates were eliminated. Following the initial article screen, 41 articles were assessed for possible eligibility. Nine articles met the specific inclusion criteria for the study and were included in the analysis (Figure 1).

Study Characteristics

A total number of nine studies were included in this review with a total of 96 patients evaluated. We identified two level II prospective studies, and seven were level IV case series and cross-sectional studies. The majority of patients in each of the studies collected were males (82%). Belhaj et al., Jain et al., and Kovacev et al. did not report on gender characteristics [10,11,15]. The age range in this study was 30-46.6 years. The mean number of weeks from injury to surgery was 77.54 ± 112.44. The follow-up period ranged from 21 to 75 months. The mean MINORS score for each of the included studies was 9.13 ± 2.23. Seven studies analyzed autograft techniques, and two studies evaluated allograft [1,3,6,7,9-11,15,16] (Table 1).

**Table 1:** Study characteristics LOE: level of evidence, M: male, F: female, MINORS: methodological index for non-randomized studies

Author	LOE	MINORS grade	Total patients	M	F	Mean age	Mean time to surgery (weeks)	Follow-up (months)	Repair technique
Abdou et al., 2014 [9]	II	10	17	14	3	30	--	21 (12-30)	Autograft
Belhaj et al., 2016 [10]	II	14	8	--	--	34.72	--	75 (29-120)	Autograft
Friedman et al., 2020 [1]	IV	9	11	8	3	46.6	34.14	54.9 (31.8-78)	Autograft
Jain et al., 2014 [15]	IV	7	9	--	--	31.5	17	54 (24-84)	Autograft
Karas et al., 2014 [16]	IV	7	11	8	3	46.48	--	52 (31-98)	Allograft
Kovacev et al., 2015 [11]	IV	9	7	--	--	42	--	48 (12-120)	Allograft
Maffulli et al., 2017 [7]	IV	--	19	16	3	46	302.43	69.6 (48-93.6)	Autograft
Sundararajan et al., 2013 [6]	IV	8	7	6	1	41.8	39.14		Autograft
Temponi et al., 2015 [3]	IV	9	7	7	0	33	69.52	41.3	Autograft

Chronic Tears: Patellar Tendon Reconstruction With Autograft

Seven out of the nine included studies (n=78 patients) reported outcomes using an autograft in their surgical technique. Six of the seven studies (n=71 patients) utilized soft tissue autografts which included the semitendinosus, gracilis, and “hamstring” tendon, while one study used a bone-tendon-bone (BTB) autograft [1,3,6,7,9,10,15]. Additionally, three of those seven studies also had the addition of augmentation, using either a suture or metal wire (McLaughlin cerclage) [1,9,10]. Moreover, the time from injury until surgery did not appear to influence the type of graft used by the authors.

Abdou et al., Friedman et al., and Belhaj et al. all utilized a soft tissue autograft with the semitendinosus tendon or hamstring tendon and added augmentation for additional stability [1,9,10]. Abdou et al. used the semitendinosus with steel wire augmentation and reported that most patients (70.6%) marked either “normal” or “nearly normal” on the postoperative International Knee Documentation Committee (IKDC). The postoperative Lysholm score was also reported to be 85. Outcomes also displayed that most patients (88.2%) had “no lack of flexion” postoperatively. The authors concluded the use of hamstring autograft provided patients with good stability and excellent outcomes [9]. Friedman et al. also used the semitendinosus in patellar reconstruction and a suture for augmentation. Postoperative flexion was 116.7 ± 12.7 degrees, extension was 0.33 ± 1.8 degrees, and extension lag was 0 degrees. It was also reported that a postoperative visual analog scale (VAS) was used to grade satisfaction with the results being 5.6 ± 3.4. It was concluded this technique is acceptable both objectively and subjectively when direct repair is not possible [1]. Finally, Belhaj utilized a hamstring tendon autograft with the addition of a metal wire (McLaughlin cerclage) for additional stability. Outcomes included postoperative data on the ROM (93.75 ± 13.56), patient satisfaction utilizing a 4-point scale (3.00 ± 1.07), EuroQol-visual analog scales (EQ-VAS) (2.63 ± 1.30), Knee Society Score (KSS) knee score of 73.13 ± 13.85, and KSS function score of 78.75 ± 21.67 [10].

Jain et al. and Sundararajan et al. each used hamstring tendons for autograft reconstruction, and Maffuli et al. used both semitendinosus and gracilis tendons. Each study reported a postoperative Insall-Salvati index with a range of 0.8-1.3 and an extension lag range of 0-3° [6,7,15]. Moreover, Jain et al. and Sundararajan et al. reported a Lysholm score range of 87-100 [6,15]. Maffuli et al. and Sundararajan et al. each reported postoperative Kujala scores with a range of 69-97 and a postoperative flexion range of 115-135. Additionally, Maffuli et al. had a postoperative Cincinnati score of 84 ± 5.7 (73-91), and Sundararajan et al. reported a postoperative IDKC of 86.8 (80-92).

Temponi et al. was the only study that described patellar tendon reconstruction using BTB autografts at a mean of 16.3 months following rupture. At the final follow-up, the mean IKDC score was 64.5, the mean Tegner score was 4, the mean Lysholm score was 79, and the mean Caton-Deschamps index was 1.2. Although no revisions were required, no patients returned to their pre-injury level of activity.

Graft Fixation

Each of the aforementioned articles utilized a patellar and tibial tuberosity tunnel as their form of fixation, except Temponi et al [1,6,7,9,10,15]. Additionally, each study used a different method for securing the graft, either a steel wire, tendon-to-periosteum stitch, 7 mm interference screws, or suturing the tendon ends [6,7,9,15]. Temponi et al. did not use tunnels and opted to fixate the graft utilizing two screws in the tibia and one screw in the patella [3] (Table 2).

**Table 2:** Surgical treatment of chronic patellar tendon ruptures with tendon graft reconstruction (autograft) ROM: range of motion, BTB: bone-tendon-bone, IKDC: International Knee Documentation Committee, VAS: visual analog scale, EQ-VAS: EuroQol-visual analog scale, KSS: Knee Society Score

Surgical treatment of chronic patellar tendon ruptures with tendon graft reconstruction (autograft)								
Autograft reconstruction								
Author (year)	Graft/technique/fixation	Augmentation	Outcomes				Conclusion	
			ROM	Functional outcomes	Complications	Postoperative mobilization		
Temponi et al., 2015 [3]	BTB autograft. Fixation: two screws in the tibia and one in the patella	--		IKDC pre-op: 45 (± 10.8). IKDC post-op: 64.5 (± 12.4). Lysholm pre-op: 45.4 (± 11.3). Lysholm post-op: 79 (± 11.8). Tegner pre-op: 1 (range 1-2). Tegner post-op: 4 (range 2-5)	--	Immediate weight-bearing in knee brace locked in extension. The knee brace was unlocked after six weeks		
							The contralateral BTB graft is a safe and viable option in the surgical treatment of chronic patellar tendon ruptures with good functional outcomes	
Abdou et al., 2014 [9]	Semitendinosus tendon autograft. Fixation: draft was passed through the tunnel in the proximal patella and tibial tuberosity secured with steel wire	Steel wire	Extension range: loss of less than 5° of extension (94.2%), loss of 6-10° of extension (5.8%). Flexion range: no lack of flexion (88.2%), lack of up to 15° of flexion (11.8%)	Lysholm: 85 (range not given). IKDC: normal (29.4 %), nearly normal (41.2%), abnormal (17.6 %), severely abnormal (11.8 %)	--	Bivalved cylinder cast for six weeks	Hamstring autograft for patellar tendon reconstruction provides good stability and excellent outcomes	
Maffulli et al., 2017 [7]	Gracilis and semitendinosus autograft. Fixation: tendon-to-periosteum for patella tunnel holes and bioabsorbable 7 mm interference screw for the distal tunnel		Post-op flexion: 132° (115-135°). Post-op extension lag: 3°	Post-op modified Cincinnati score: 84 ± 5.7 (73-91). Post-op Kujala score: 81 ± 6.1 (69-91). Post-op Insall-Salvati index: 1.5 (1.2-1.8)	1 – lateral portion of patella partially breached while drilling 1 – tibial tuberosity partially detached	Leg immobilized in full extension	The method of choice is early surgical intervention. Comorbidity and risk factors can lead to a poor postoperative Lysholm score	
Sundararajan et al. 2013 [6]	Hamstring tendon autograft. Fixation: transverse bone tunnels in the lower half of the patella and tibial tuberosity. Tendon ends sutured by 2-0 Ethibond		Post-op flexion: 125° (range 120–130°). Post-op extension lag: 0	Post-op Lysholm: 92.4 (range 89-95). Post-op IKDC: 86.8 (range 80-92). Post-op Kujala score: 94.5 (range 92–97). Post-op Insall-Salvati index: 1.08 (range 0.8-1.3)			Reconstruction of neglected patellar tendon ruptures utilizing hamstring autografting provides good stability and excellent outcomes	
Jain et al., 2014 [15]	Semitendinosus autograft (percutaneous). Fixation: patella tunnel and tibial tunnel. The graft was sutured together		Post-op extension lag: 0°	Post-op Lysholm: 94.44 (87-100). Post-op Insall-Salvati index: 1.16 (1.12-1.30)	--	Knee brace locked in extension for six weeks	Excellent results with a low rate of complications using direct suture repair and wire cerclage followed by early mobilization	
Friedman et al., 2020 [1]	Semitendinosus autograft. Fixation: tunnels in the patella and tibial tuberosity with anterolateral tibia docking utilizing an anchor	Suture	Post-op flexion: 116.7° ± 12.7°. Post-op extension 0.33° ± 1.8°. Post-op extension lag: 0°	Post-op VAS (satisfaction): 5.6 ± 3.4			Percutaneous reconstruction showed to be beneficial in reducing morbidity. Also, a percutaneous approach diminishes the need for preoperative traction and second surgery for the removal of cerclage wire	
Belhaj et al., 2016 [10]	Hamstring tendon autograft. Fixation: two bone tunnels	McLaughlin cerclage	Post-op ROM: 93.75 ± 13.56	Post-op satisfaction (1-4 scale): 3.00 ± 1.07. Post-op EQ-VAS: 2.63 ± 1.30. Post-op KSS-knee: 73.13 ± 13.85. Post-op KSS function: 78.75 ± 21.67	--	Closed cylinder cast for six weeks	Long-term follow-up showed excellent results with early surgical repair being ideal for patella tendon ruptures. Chronic patella tendon ruptures may require a longer rehabilitation protocol	
Notes: Abbreviations: ROM: range of motion, BTB: bone-tendon-bone, IKDC: International Knee Documentation Committee, VAS: visual analog scale, EQ-VAS: EuroQol-visual analog scale, KSS: Knee Society Score								

Chronic Tears: Patellar Tendon Reconstruction With Allograft

Two studies used allograft reconstruction as part of their technique. Karas et al. reported outcomes for Achilles allograft and whole BTB allograft, while Kovacev et al. reported outcomes for BTB allograft only [11,16]. The postoperative Lysholm score range for both studies was 28-100. Karas et al. also described a postoperative flexion of 130 degrees, extension lag of 8 degrees (0-18), IDKC of 74 (28-90), and Tegner of 8 (0.5-10). Karas et al. utilized a proximal dovetail stitch and two distal 3.5 mm AO screws with a no. 5 nonabsorbable suture formed in a Krackow fashion for graft fixation (Table 3).

**Table 3:** Surgical treatment of chronic patellar tendon ruptures with tendon graft reconstruction (allograft) ROM: range of motion, BTB: bone-tendon-bone, IKDC: International Knee Documentation Committee

Surgical treatment of chronic patellar tendon ruptures with tendon graft reconstruction (allograft)						
Allograft reconstruction						
Author (year)	Graft/technique/fixation	Outcomes				Conclusion
		ROM	Functional outcomes	Complications	Postoperative mobilization	
Karas et al., 2014 [16]	11/14: Achilles allograft. 3/14: whole BTB allograft. Fixation: press fit into the site on the patella and dovetail stitched. Distally, calcaneal bone block press fit with two 3.5 mm AO screws and no. 5 non-absorbable sutures woven in a Krackow fashion	Post-op flexion: 130°. Post-op extension lag: 8° (0-18°)	Post-op Lysholm: 62 (28-100). Post-op IKDC: 74 (28-90). Post-op Tegner: 8 (0.5-10)		Hinged knee brace locked in extension for first six weeks	This procedure was shown to decrease pain and increase the ability to perform activities of daily living. However, it also proved to result in “moderate” patient satisfaction and may not return patients to high-level activity
Kovacev et al., 2015 [11]	BTB allograft fixation: not mentioned		Post-op Lysholm: 67	Quadriceps atrophy	Immobilized for six weeks	
Notes: Abbreviations: BTB: Bone-Tendon-Bone, ROM: Range of motion, IKDC: International Knee Documentation Committee						

Discussion

This is the largest and most comprehensive systematic review to date which evaluated outcomes and complications following chronic patellar tendon reconstruction. In the current literature, acute patellar tendon repairs have been shown to have both good postoperative functional outcomes and a low rate of complications. This study showed somewhat similar outcomes (fair to good) following chronic patellar reconstruction while utilizing either autografts or allografts. The overall findings from this study show positive postoperative functional outcome results from patellar tendon reconstruction. These include increased Lysholm scores from preoperative to postoperative and a decrease in VAS pain score [1,3,6,9,11,15,16]. Furthermore, besides intraoperative complications such as the lateral portion of the patella being breached while drilling and the tibial tuberosity becoming partially detached, there were no major postoperative complications resulting in graft failure [7]. Additionally, the postoperative ROM was restored in each study and an observed extensor lag following the reconstruction was minimal [7,16]. Overall, this study shows reconstruction utilizing allograft and autograft with or without augmentation can lead to positive functional outcomes, revival of the extensor mechanism and ROM, and decreased complications.

Graft selection is important when performing any ligament or tendon reconstruction as the biomechanical properties of the graft can significantly affect the strength and incorporation of the graft. The most commonly chosen graft in this systematic review was the autograft semitendinosus tendon. Semitendinosus autograft is commonly chosen for many tendon reconstruction procedures and has shown to be advantageous in patellar tendon repairs. Reconstruction with the semitendinosus has been shown in cadaver studies to decrease gap formation with cyclic loading [17]. This is important to consider since gap formation can lead to re-ruptures, knee stiffness, and extensor lag [17]. Additionally, three studies analyzed the use of autograft along with augmentation. The use of augmentation is thought to decrease strain across the primary repair site which can assist in preventing re-rupture [18]. Each study showed good postoperative outcomes; however, it was noted that earlier surgical intervention would have been a more ideal treatment strategy and may have further optimized outcomes [10].

In addition to the use of autografts, the use of allografts was also examined in this systematic review. The use of BTB allografts had been previously studied in case reports and patellar tendon reconstruction following total knee arthroplasty [19-22]. It was shown that a BTB allograft was a successful salvage procedure for patients that were unable to have their patella tendon rupture repaired in a short time frame. Moreover, there are advantages to utilizing BTB allografts such as bone-to-bone healing, lack of donor site morbidity, and a large amount of tissue available for reconstruction [23]. As shown in this systematic review, BTB allograft provided fair postoperative functional outcomes; however, there are concerns about returning to previous levels for sports [16]. While allograft reconstruction seems to provide fair postoperative results, overall, allograft had subjectively worse patient clinical outcomes when compared to autograft use in this study. Prior studies that have utilized allografts to reconstruct chronic patellar tendon ruptures show fair results with potential for major complications such as recurrent extensor mechanism disruption and postoperative extensor lag [24-27]. The worse outcomes seen with allografts are likely a result of the chronicity and proximal migration of the patella over time, which makes allograft the most suitable option. As a result, there is less likelihood of adequate healing and the subsequent patella alta translates into worse clinical outcomes [28].

Postoperative management for chronic patellar tendon rupture reconstruction was similar between the autograft and allograft groups. Each study implemented a postoperative protocol that involved immobilization in either a cylinder cast or a hinged knee brace locked in extension. The rationale to limit flexion in the initial six weeks following surgery is to allow for graft healing and limit elongation (creep). Flexion will increase the stretch of the graft, and stress will be transmitted at the sites of attachment to the bone and can influence graft pull-out. Additionally, grafts progress through stages of healing and incorporation into their new donor site [27]. Therefore, optimal postoperative rehabilitation for chronic patellar tendon rupture repair utilized with autograft or allograft should have an initial immobilization phase to protect the graft. For BTB autografts, since they retain the native tendon-bone junction, the primary healing is bone-to-bone [8]. Since the healing is between bones, it has been shown to heal more rapidly in laboratory studies. Prolonged immobilization can lead to decreased patellar mobility and muscle weakness. Therefore, after a proper course of immobilization, the addition of leg motion should be encouraged to decrease adhesion formation and promote healthy cartilage formation [28].

Strengths of this systematic review include a comprehensive search strategy and rigorous data collection, which resulted in a large cohort of patients for a relatively rare injury. A total of 96 patients and their outcomes following chronic patellar reconstruction were analyzed, while most individual studies are restricted to case reports or small case series. This study also reports on postoperative rehabilitation protocols and optimal postoperative care to allow proper healing of the graft, which has not been previously reviewed in this manner. Limitations to the study include the inherently rare nature of the pathology. Most patients can obtain a patellar tendon repair acutely to restore the function of the extensor mechanism. Therefore, there is a paucity of literature and high-quality articles that analyze chronic patellar tendon repairs. Additionally, due to the heterogeneity of the reconstructive techniques used and functional outcome assessment tools measured, this study was unable to proceed with a meta-analysis of the data and had qualitative report outcomes.

## Conclusions

While chronic patellar tendon ruptures are a rare injury of the extensor mechanism, there are viable options for reconstruction. Overall, chronic patellar tendon ruptures reconstructed with both autograft and allograft will provide fair to good outcomes with low complication rates. Following surgery, immobilization for at least six weeks should be emphasized to protect the graft. Further high-level studies are needed to determine if certain techniques are more optimal for reconstruction.
